# Insulin Resistance and Atherogenic Dyslipidemia Drive Cardiac Remodeling and Cardiovascular Events After Kidney Transplantation

**DOI:** 10.3390/jcm15082915

**Published:** 2026-04-11

**Authors:** Ioana Adela Ratiu, Cristina Mihaela Brisc, Alina Daciana Elec, Corina Moisa, Anamaria Ratiu, Edy Hagi-Islai, Cristian Adrian Ratiu, Ioana Paula Blaj-Tunduc, Victor Vlad Babeș, Emilia Elena Babeș

**Affiliations:** 1 Department of Medical Disciplines, Faculty of Medicine and Pharmacy, University of Oradea, 1st December Square 10, 410073 Oradea, Romania; ioana.ratiu@didactic.uoradea.ro (I.A.R.); brisccristina@uoradea.ro (C.M.B.); eebabes@uoradea.ro (E.E.B.); 2 Nephrology Department, Emergency Clinical Hospital Bihor County, 12 Corneliu Coposu Street, 410469 Oradea, Romania; 3 Clinical Institute of Urology and Renal Transplantation, 400006 Cluj-Napoca, Romania; alina.elec@icutr.ro; 4 Department of Pharmacy, Faculty of Medicine and Pharmacy, University of Oradea, 1st December Square 10, 410073 Oradea, Romania; 5 Faculty of Dentistry, University of Medicine and Pharmacy “Iuliu Hatieganu” Cluj-Napoca, Victor Babeș Street 8, 400347 Cluj-Napoca, Romania; ratiu.anamaria@elearn.umfcluj.ro (A.R.); hagi.islai.edy@elearn.umfcluj.ro (E.H.-I.); 6 Discipline of Oral Implantology, Dentistry Department, Faculty of Medicine and Pharmacy, University of Oradea, 1st December Square 10, 410073 Oradea, Romania; 7 Doctoral School of Biomedical Sciences, Faculty of Medicine and Pharmacy, University of Oradea, 1st December Square 10, 410073 Oradea, Romania; blaj.ioanapaula@student.uoradea.ro; 8 Cardiology Department, Emergency Clinical Hospital Bihor County, 65 Gheorghe Doja Street, 410169 Oradea, Romania; vvbabes@uoradea.ro

**Keywords:** kidney transplant, insulin resistance, atherogenic index of plasma, triglyceride-glucose (TyG) index, echocardiographic damage composite, MACO—major adverse clinical outcome, MACCE—major adverse cerebro-cardiovascular events, neutrophil-to-lymphocyte ratio (NLR), CRP/albumin ratio and NLR/albumin ratio, platelet-to-lymphocyte ratio (PLR)

## Abstract

**Background**: Cardiovascular disease remains a leading cause of morbidity and mortality after kidney transplantation. The relative contribution of metabolic abnormalities and inflammatory burden to cardiac remodeling and subsequent clinical outcomes in kidney transplant recipients (KTRs) remains incompletely understood. **Methods**: In this retrospective cohort study, 152 KTRs underwent comprehensive cardiovascular evaluation at a stable post-transplant time point (12 ± 4 months after transplantation). Metabolic phenotype was assessed using metabolic syndrome and indices of insulin resistance and atherogenic dyslipidemia (TyG index, TG/HDL ratio, and atherogenic index of plasma [AIP]). Inflammatory status was evaluated using hs-CRP and complete blood count-derived indices. Echocardiographic damage composite (EDC) was defined as the presence of left ventricular hypertrophy, diastolic dysfunction, or left atrial enlargement. Patients were followed for major adverse clinical outcome (MACO), defined as cardiovascular event, graft failure, or death, and major adverse cardiovascular and cerebrovascular events (MACCE). **Results**: At baseline, 78 patients (51.3%) met criteria for EDC. EDC was strongly associated with higher TyG, AIP, TG/HDL, LDL/HDL ratio, and metabolic syndrome, whereas inflammatory markers showed no association. In multivariable logistic regression adjusted for age, sex, eGFR, and proteinuria, TyG remained independently associated with EDC (OR 1.13 per 0.1 increase, 95% CI 1.05–1.21; *p* = 0.001), independent of hs-CRP. Similar results were observed when AIP was evaluated in place of TyG (OR 10.39, 95% CI 2.22–48.71; *p* = 0.003). During follow-up, 78 patients developed MACO and 49 developed MACCE. In Cox regression analysis, graft dysfunction and inflammatory markers independently predicted MACO, whereas TyG was no longer significant. In contrast, TyG remained an independent predictor of MACCE after adjustment for confounders and inflammatory markers (HR 1.10 per 0.1 increase, 95% CI 1.04–1.16; *p* < 0.001). Similar results were observed when AIP was tested in place of TyG (HR 10.8, 95% CI 3.06–38.11; *p* < 0.001). Echocardiographic damage did not independently predict outcomes after adjustment. **Conclusions**: In KTRs, metabolic abnormalities reflecting insulin resistance and atherogenic dyslipidemia are closely associated with cardiac remodeling one year after transplantation and remain specifically linked to subsequent cardiovascular events. In contrast, systemic inflammation and graft dysfunction are the primary determinants of overall adverse clinical outcomes. Simple metabolic indices such as TyG and AIP may provide practical tools for cardiovascular risk stratification in this population. In Cox proportional hazards models, TyG (HR 1.102, 95% CI 1.043–1.164, *p* = 0.001) and AIP (HR 10.8, 95% CI 3.06–38.11, *p* < 0.001) were independently associated with cardiovascular events during follow-up, underscoring the role of atherogenic dyslipidemia in cardiovascular risk.

## 1. Introduction

Kidney transplantation is currently considered the optimal modality of renal replacement therapy. Nevertheless, its prevalence remains significantly lower than that of dialysis [1,2]. In Europe, kidney transplantation accounts for approximately 6% of all renal replacement therapy modalities, with wide variation across geographic regions and patient age [3]. Patient and graft survival are higher with living-donor kidney transplants than with deceased donors. In Europe, five-year patient survival is 95.2% vs. 91.6%, and graft survival is 88% vs. 80.6%, favoring living donation [3]. To address donor shortages, graft acceptance has increased, and immunosuppressive regimens have evolved to balance efficacy and individualization. As a result, infection is now the main post-transplant complication. The pandemic marked a paradigm shift in kidney transplant recipients (KTRs), as in hemodialysis patients, with COVID-19 deaths rising exponentially [4]. Since then, infections have remained the leading cause of death, followed by neoplastic and cardiovascular diseases. In the United States, among deaths with a known cause post-kidney transplant, 8% were attributed to cardiovascular pathology [1].

**Figure 1:**
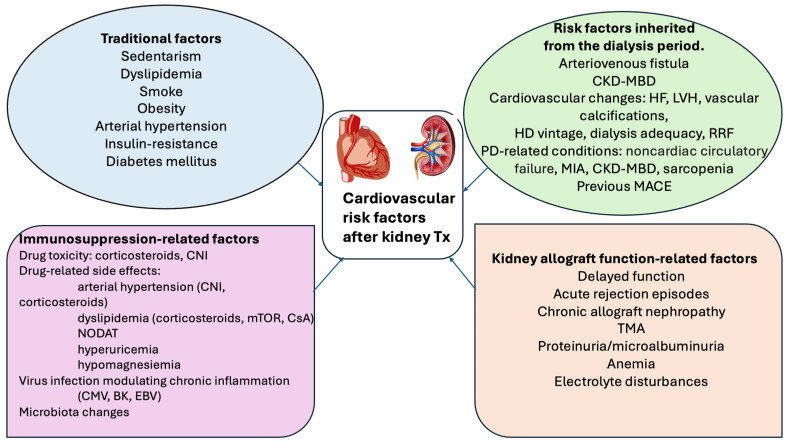
Cardiovascular Risk Factors Following Renal Transplantation. Legend: Tx—transplantation; CNI—calcineurin inhibitors; CsA—ciclosporin A; NODAT—new onset diabetes after transplantation; CMV—cytomegalovirus; BK—polyoma BK virus; EBV—Epstein–Barr virus; CKD-MBD—chronic kidney disease mineral bone disease, LVH—left ventricular hypertrophy; MACE—major adverse cardiovascular events; RRF—residual renal function; PD—peritoneal dialysis; MIA—malnutrition–inflammation–atherosclerosis; TMA—thrombotic microangiopathy [14].

Like in HD, cardiovascular disease remains one of the leading causes of morbidity and mortality after kidney transplantation, despite the restoration of kidney function [5,6]. In the post-transplant setting, multiple cardiovascular risk factors coexist, including traditional factors as well as transplant-specific factors like immunosuppressive therapy and graft nephropathy. Therefore, the kidney transplant recipient is a clinically “upgraded” former dialysis patient who still bears irreversible consequences of prior end-stage renal disease (ESRD). Residual risk factors from the ESRD period continue to affect outcomes after transplantation, including mineral and bone disease, dialysis vintage, persistent high-flow arteriovenous fistulas, vascular stenoses related to prolonged central venous catheter use, and the burden of pre-transplant cardiovascular events [7,8]. Traditional risk factors common in KTRs, such as obesity, dyslipidemia, diabetes, smoking, and hypertension, are closely linked to impaired insulin sensitivity. Reduced renal allograft function and the metabolic effects of immunosuppressive therapy further contribute to the high prevalence of insulin resistance after transplantation [9,10]. Metabolic syndrome, a condition strongly associated with insulin resistance, affects nearly one-third of KTRs within the first-year post-transplant and is a known predictor of cardiovascular events [11,12]. Obesity has an incidence exceeding 33% after renal transplantation, representing another link in the chain contributing to increased cardiovascular risk [11]. Furthermore, the use of calcineurin inhibitors as first-line immunosuppressive therapy after transplantation is associated with a significant incidence of hypomagnesemia, which is an important contributor to cardiovascular risk through complex mechanisms involving insulin resistance, amplification of the pro-inflammatory state, worsening of dyslipidemia, endothelial dysfunction, arterial hypertension, and atherogenesis [13] (Figure 1).

Currently, three lines of immunosuppression are used to maintain renal graft viability post-transplant: calcineurin inhibitors (CNIs) (cyclosporine and tacrolimus), purine synthesis inhibitors (mycophenolate mofetil and azathioprine), and corticosteroids. These drugs increase post-transplant cardiovascular risk through multiple mechanisms: dyslipidemia (more pronounced with cyclosporine), arterial hypertension (cyclosporine and corticosteroids), diabetes mellitus (corticosteroids and tacrolimus), and cardiac remodeling and fibrosis (CNIs and corticosteroids). mTOR inhibitors, whose use is generally limited to cases of CNI toxicity, intolerance, or the presence of neoplastic disease, induce dyslipidemia and proteinuria, further increasing cardiovascular risk [7]. An alternative in this context has proven to be the use of belatacept, which does not induce dyslipidemia or diabetes mellitus and has no adverse cardiovascular effects. It is particularly useful in patients with high cardiovascular risk, although careful monitoring is required due to an increased risk of infections [15]. Likewise, therapeutic strategies involving early corticosteroid withdrawal in selected patient subgroups have been shown to benefit cardiovascular protection, although the risk of renal graft loss remains high [16].

Kidney transplantation mitigates the severity of several cardiovascular risk factors associated with dialysis, partly by improving objective parameters such as maximal oxygen consumption, regressing left ventricular hypertrophy, and enhancing ejection fraction [17]. However, atrial fibrillation that persists from the dialysis period remains associated with an increased risk of post-transplant stroke, while persistent brachiocephalic arteriovenous fistulas may contribute to the development of pulmonary hypertension. The spectrum of cardiovascular disease in KTRs largely mirrors that observed in the chronic kidney disease population and includes coronary artery disease, valvular heart disease, heart failure, stroke, cardiac arrhythmias, pulmonary hypertension, and peripheral arterial disease [18].

Post-transplant, pre-existing cardiovascular alterations coexist with residual graft dysfunction and chronic low-grade inflammation, creating a complex cardiometabolic environment that may drive progressive cardiovascular injury [19,20]. This constellation is increasingly conceptualized as the cardio–reno–metabolic syndrome, characterized by the interplay of cardiac, renal, and metabolic dysfunction, with systemic inflammation serving as a central determinant of both pathogenesis and prognosis [21]. Under these conditions, assessing post-transplant cardiovascular risk factors is particularly complex and nuanced [22]. Echocardiographic abnormalities such as left ventricular hypertrophy, diastolic dysfunction, and left atrial enlargement are highly prevalent in KTRs and represent early manifestations of cardiovascular involvement. However, the relative contributions of metabolic abnormalities and inflammatory burden to this cardiac remodeling in the stable post-transplant phase remain insufficiently understood. Moreover, it is unclear whether the same factors associated with subclinical cardiac damage are also linked to subsequent adverse clinical outcomes.

Simple indices derived from routine laboratory tests, such as the triglyceride-glucose (TyG) index and the Atherogenic Index of Plasma (AIP), have emerged as reliable markers of insulin resistance and atherogenic dyslipidemia and may provide a more sensitive characterization of cardiometabolic risk than the conventional definition of metabolic syndrome. The TyG index has become a prominent and reliable indicator of insulin resistance, often surpassing traditional metrics such as the HOMA-IR index in several studies [23]. Furthermore, numerous studies have linked the TyG index to cardiovascular diseases and mortality, emphasizing its relevance across various cohorts [24,25,26,27,28], including those suffering from chronic kidney disease [29,30].

The atherogenic index of plasma (AIP), introduced by Dobiasova and Frohlich in 2001, is calculated as the logarithm of the triglyceride-to-HDL cholesterol ratio and provides insights into disturbances in lipid metabolism [31]. A growing body of research underscores AIP’s role as a predictor of cardiovascular disease risk [32,33], though its significance in chronic kidney disease and KTRs is less clearly established. While cross-sectional studies have demonstrated associations between higher AIP values and albuminuria and reduced glomerular filtration rate, longitudinal data remain scarce [34,35,36] AIP might contribute to renal injury through mechanisms related to endothelial dysfunction, oxidative stress, and inflammation [37].

The immune system links inflammation and dyslipidemia, contributing to atherogenesis and endothelial injury in CKD patients [38,39].

High-sensitivity CRP (hs-CRP), a component of the innate immune system, is a reliable biomarker of cardiovascular risk in kidney transplant patients. It promotes endothelial injury, oxidative stress, platelet aggregation, and phagocyte activation [40,41]. At the same time, inflammatory markers derived from hs-CRP and complete blood count indices are frequently used as surrogates of systemic inflammation in transplant recipients. The main inflammatory biomarkers that have been correlated with increased cardiovascular mortality and progression of renal disease in the general population are the systemic inflammatory index (SII), the high-sensitivity C-reactive protein/HDL cholesterol ratio, the systemic immune-inflammation index, and the dietary inflammation index [42,43].

As an expression of the imbalance between innate and adaptive immunity, the neutrophil-to-lymphocyte ratio (NLR) has been shown to predict renal disease progression in patients with chronic kidney disease (CKD) [44]. It has also been shown to act as a marker of disease severity and adverse prognosis in the general heart failure population, including acute myocarditis, and to predict both cardiovascular and all-cause mortality [45,46,47,48]. Some studies have shown a correlation between NLR and the new onset or recurrence of atrial fibrillation [49]. Through their pro-oxidant and proteolytic effects, neutrophils can exacerbate endothelial injury, amplifying renal and cardiovascular damage post-transplant. However, studies specifically addressing the post-transplant patient population are scarce in the literature [50,51].

In this context, the aim of this study was twofold: first, to evaluate the relationship between metabolic phenotype and inflammatory status with echocardiographic cardiac remodeling in KTRs during a stable post-transplant phase; and second, to determine whether these baseline characteristics are associated with subsequent adverse clinical outcomes, including cardiovascular events, graft failure, and mortality.

Unlike previous studies conducted early after transplantation or focusing solely on structural cardiac changes or clinical outcomes, this study evaluates cardiometabolic and inflammatory profiles at a physiologically stable time point and examines their differential associations with both subclinical cardiac remodeling and longitudinal clinical evolution.

## 2. Materials and Methods

### 2.1. Study Design and Population

We conducted a retrospective cohort study with a landmark time-to-event design, including 152 adult KTRs followed at the Nephrology Department of the County Emergency Clinical Hospital Bihor between January 2015 and December 2024. From the baseline, patients were followed for major adverse events after transplantation (Tx). The data were collected from hospitalization records, outpatient documentation, and electronic medical follow-up records. The study was conducted in accordance with the Declaration of Helsinki and received approval from the hospital’s ethics committee (nr. 3978/06.02.2025).

**Inclusion Criteria**

(a) adult patients (≥18 years old); (b) history of kidney transplantation performed between 2015 and 2024; (c) functioning renal graft at 12 months post-transplantation; (d) availability of complete clinical, laboratory, and echocardiographic data at approximately 12 months post-transplant (T0); (e) minimum follow-up duration of 3 months after T0.

**Exclusion Criteria**

**Figure 2:**
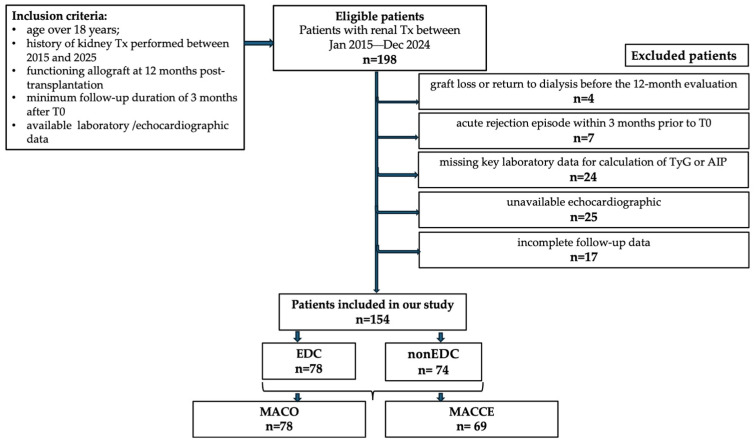
Patients’ flowchart. Legends: Tx—transplantation; EDC—Echocardiographic Damage Composite; MACO—Major Adverse Clinical Outcome; MACCE—Major Adverse Cerebro-Cardiovascular Events.

(a) graft loss or return to dialysis before the 12-month evaluation; (b) acute rejection episode within 3 months prior to T0; (c) missing key laboratory parameters required for calculation of TyG or AIP; (d) incomplete follow-up data; (e) poor-quality or unavailable echocardiographic assessment (Figure 2).

All kidney transplant candidates underwent a standardized pre-transplant evaluation in accordance with national transplant protocols. Clinical, imaging, and laboratory investigations were performed to exclude active infection, malignancy, and comorbid conditions constituting contraindications to Tx. Particular attention was paid to cardiovascular assessment to identify clinically significant coronary artery disease, heart failure, or other major cardiovascular disorders.

Mandatory cardiovascular evaluation included a 12-lead electrocardiogram and transthoracic echocardiography. When clinically indicated, further noninvasive testing, such as exercise stress testing, dobutamine stress echocardiography, or cardiac computed tomography, was performed. Cardiological reassessment was repeated annually while patients remained on the transplant waiting list.

Therefore, at the time of Tx, candidates included in this study had undergone systematic cardiovascular screening and were free of clinically significant overt cardiovascular disease. This selection process explains the predominantly preserved systolic function observed in the cohort and supports the interpretation that echocardiographic abnormalities identified after Tx reflect persistence or incomplete regression of CKD-related diastolic remodeling rather than pre-existing overt cardiovascular disease.

We defined baseline (T0) as the first comprehensive cardiovascular evaluation performed after kidney Tx, occurring at a median of approximately 12 ± 4 months post-transplant. This time point was deliberately selected to represent a stable post-transplant phase after resolution of early peri-transplant hemodynamic, inflammatory, and metabolic fluctuations. Patients were included if complete metabolic, inflammatory, and echocardiographic data were available at the time of this visit.

### 2.2. Data Collection at Baseline

The following baseline data were recorded at the 1-year postTx landmark:

(a) **Demographics:** age, gender, Tx vintage;
(b) **Biometrics:** BMI, BSA;
(c) **Lifestyle:** smoking;
(d) **Type of immunosuppressive treatment**: CNI (tacrolimus or ciclosporin), corticosteroids, mTOR (rapamycin);
(e) **Other treatments**: statins, beta-blockers;
(f) **Comorbidities:** malignancies, arterial hypertension, diabetes;
(g) **Assessment of metabolic phenotype.**

Metabolic status was evaluated using:

- Metabolic syndrome was considered present when at least three metabolic abnormalities were identified, according to the NCEP ATP III framework. These included abdominal obesity (waist circumference >102 cm in men or >88 cm in women), elevated triglyceride concentrations (≥150 mg/dL), low HDL cholesterol levels (<40 mg/dL in men or <50 mg/dL in women), increased blood pressure (≥130/85 mmHg or ongoing antihypertensive treatment), and impaired fasting glucose (≥100 mg/dL) [52].
- Triglycerides (TG), HDL-cholesterol, LDL-cholesterol
- TG/HDL-cholesterol ratio
- LDL/HDL-cholesterol ratio
- **TyG index**, calculated as: ln (Triglycerides (mg/dL) ×Fasting glucose (mg/dL)/2)
- **Atherogenic Index of Plasma (AIP)**, calculated as log (TG/HDL cholesterol); values for triglycerides and HDL cholesterol were converted from **mg/dL** to **mmol/L** (TG (mg/dL) × 0.0113 = mmol/L; HDL-cholesterol (mg/dL) × 0.0259 = mmol/L)

(h) **Assessment of inflammatory status**

Inflammatory burden was assessed using:

- High-sensitivity C-reactive protein (hs-CRP)
- Neutrophil-to-lymphocyte ratio (NLR)
- CRP/albumin ratio and NLR/albumin ratio
- Platelet-to-lymphocyte ratio (PLR)

Biochemical parameter triglycerides, total cholesterol, LDL cholesterol, HDL cholesterol, glucose, creatinine, magnesium, CRP, and albumin were determined using the Abbott ALINITY c analyzer (AC06028) (Abbott Laboratories, North Chicago, IL, USA) and chemiluminescent microparticle immunoassay technique (CMIA). The complete blood cell count was performed using CMIA on the Abbott Alinity hq (HQ00687) analyzer. NTproBNP was measured using the CMIA technique on the Abbott Alinity C analyzer (AC03837).

**Echocardiographic evaluation and definition of cardiac damage**

Echocardiography was performed according to recommendations of the American Society of Echocardiography (ASE) and the European Association of Cardiovascular Imaging (EACVI). Diastolic function was assessed using a combination of guideline-recommended echocardiographic indices, including mitral annular e′ velocity, trans mitral E/A ratio, average E/e′ ratio, and left atrial volume index (LAVI).

Impaired relaxation was defined by reduced mitral annular e′ velocity. Diastolic dysfunction was considered present when reduced e′ velocity (<6.5 cm/s) was accompanied by at least one additional abnormal parameter reflecting elevated left ventricular filling pressure (average E/e′ > 14, E/A < 0.8 or ≥2, or LAVI > 34 mL/m^2^). In the presence of a normal e′ velocity, two of the above abnormal parameters were required. Left atrial volume was measured according to ASE/EACVI recommendations and indexed to body surface area [53]. Left ventricular mass was calculated using the Devereux formula and indexed to body surface area [54]. Echocardiography was performed on a LOGIQUE P7 ultrasound system, General Electric Healthcare, Chicago, IL, USA. An **Echocardiographic Damage Composite (EDC)** representing structural and functional remodeling was defined as the presence of at least one of the following:

- Left ventricular hypertrophy (LV mass index > 115 g/m^2^ in men, >95 g/m^2^ in women)
- Diastolic dysfunction
- Left atrial enlargement (left atrial volume index-LAVI > 34 mL/m^2^)

An **EDC severity score (0–3)** was calculated by summing the number of abnormalities present.

Systolic function parameters were not included in the definition of the EDC for several reasons. First, all patients underwent rigorous cardiovascular screening prior to Tx, which excludes individuals with advanced systolic dysfunction. Accordingly, LVEF was preserved in the vast majority of our cohort, resulting in limited variability and low discriminative value for identifying cardiac remodeling at the baseline evaluation. Second, the cardiac phenotype in KTRs is predominantly characterized by diastolic dysfunction, left ventricular hypertrophy, and left atrial enlargement, consistent with heart failure with a preserved ejection fraction-like pattern. These structural and functional alterations are more sensitive markers of chronic pressure and volume overload and have been more consistently associated with adverse outcomes in CKD and transplant populations than systolic indices. In this context, LVEF is not a sensitive marker of early myocardial remodeling and may remain normal despite significant structural and functional abnormalities. Third, the aim of the EDC was to capture subclinical cardiac remodeling rather than overt systolic dysfunction. In this context, diastolic and structural parameters were considered more appropriate, as they reflect early myocardial changes that may persist after Tx despite preserved ejection fraction.

### 2.3. Clinical Outcomes During Follow-Up

Patients were followed from baseline through last contact for the occurrence of:

- **Major Adverse Clinical Outcome (MACO):** a combined endpoint including the first occurrence of cardiovascular event, graft failure requiring return to dialysis, or death from any cause.

Graft failure was defined as return to chronic dialysis therapy or re-transplantation due to loss of kidney graft function.

- **Major Adverse Cardiovascular and cerebrovascular Events (MACCE):** a combined endpoint including: coronary artery disease, heart failure, atrial fibrillation, peripheral arterial disease, stroke, or cardiovascular death.

Cardiovascular death was defined as death resulting from acute myocardial infarction, heart failure, stroke, fatal arrhythmia, sudden cardiac death, or other documented cardiovascular cause as recorded in the medical file or death certificate.

Coronary artery disease (CAD) was defined as a documented history of myocardial infarction (with or without ST-segment elevation), percutaneous coronary intervention (PCI) or coronary artery bypass grafting (CABG), or angiographically confirmed coronary stenosis ≥ 50%. Hospitalization for unstable angina accompanied by documented objective evidence of myocardial ischemia (ischemic ECG changes, new regional wall motion abnormalities on echocardiography, positive stress testing, or angiographic evidence of significant coronary stenosis) was also considered CAD.

Heart failure (HF) was defined as a documented clinical diagnosis of heart failure requiring hospitalization or initiation/intensification of diuretic therapy, accompanied by objective evidence of cardiac dysfunction, including reduced or preserved left ventricular ejection fraction with signs of congestion, elevated natriuretic peptides, or imaging evidence of cardiac dysfunction.

Atrial fibrillation (AF) was defined as a documented episode of atrial fibrillation on electrocardiogram (ECG), Holter monitoring, or hospital record, including paroxysmal, persistent, or permanent AF.

Peripheral arterial disease (PAD) was defined as a documented diagnosis of lower extremity arterial disease in the medical record, supported by imaging evidence (Doppler ultrasound, CT angiography, or conventional angiography), a history of a peripheral revascularization procedure, or an ankle–brachial index (ABI) < 0.90.

Stroke was defined as a documented acute neurological deficit lasting more than 24 h, confirmed by neuroimaging (CT or MRI) consistent with ischemic or hemorrhagic cerebral infarction.

**Statistical analysis**

Continuous variables were expressed as mean ± standard deviation and compared using t-tests or ANOVA. Categorical variables are presented as counts (percentages) and were compared using chi-square tests. The cross-sectional association between metabolic indices and echocardiographic damage composite (EDC) was first explored using univariable comparisons between patients with and without EDC.

Multivariable logistic regression models were constructed to evaluate whether metabolic and inflammatory parameters were independently associated with EDC after adjustment for clinically relevant confounders. Variable selection was guided by biological relevance, clinical interpretability, and avoidance of collinearity, rather than solely by univariable statistical significance.

The metabolic phenotype was primarily characterized using TyG and AIP, which were selected as integrated markers of insulin resistance and atherogenic dyslipidemia, capturing the combined effects of glucose and lipid metabolism more comprehensively than individual lipid parameters do.

Systemic inflammation was primarily represented by hs-CRP, chosen as the main inflammatory covariate due to its established clinical validity and widespread use as a marker of systemic inflammatory burden. Additional inflammatory indices (including NLR, NLR/albumin, and CRP/albumin) were evaluated as complementary markers in secondary analyses but were not included in the primary models simultaneously to avoid redundancy and collinearity.

EDC severity was quantified as the number of abnormalities present (0–3), and relationships between EDC severity and TyG/AIP were evaluated using one-way ANOVA with Bonferroni post hoc tests.

The discriminative performance of TyG and AIP for EDC was assessed using receiver operating characteristic (ROC) analysis. Optimal cut-off values were determined using the Youden index, and sensitivity/specificity were reported. Multivariable logistic regression models were constructed to evaluate independent associations with EDC.

For longitudinal follow-up analyses, Kaplan–Meier survival curves and Cox proportional hazards models were used to evaluate predictors of MACO and MACCE. Multivariable Cox proportional hazards models were constructed to determine whether baseline metabolic phenotype, inflammatory status, or echocardiographic damage independently predicted clinical outcomes during follow-up. Variables were selected based on biological relevance, clinical interpretability, and avoidance of collinearity, consistent with the approach used in the cross-sectional analyses.

Optimal cut-off values for TyG and AIP were determined using receiver operating characteristic (ROC) analysis with the Youden index. Because hs-CRP exhibited a skewed distribution, patients were categorized according to the median hs-CRP value (4.5 mg/L) for Kaplan–Meier survival analyses. A *p*-value < 0.05 was considered statistically significant. Analyses were performed using SPSS version 25 (IBM Corp., Armonk, NY, USA) and MedCalc statistical software v22.00.

## 3. Results

### 3.1. Baseline Characteristics of the Cohort

The study cohort comprised 152 KTRs evaluated at a predefined baseline visit (12 months ± 4 after transplantation), at which complete metabolic, inflammatory, and echocardiographic data were available for all patients. At this time point, 78 patients (51.3%) fulfilled criteria for the echocardiographic damage composite (EDC), while 74 did not (Table 1).

Patients with EDC had significantly lower graft function and higher proteinuria compared with those without EDC (eGFR: 40.7 ± 23.5 vs. 50.0 ± 26.2 mL/min, *p* = 0.02; proteinuria: 1127 ± 1513 vs. 672 ± 933 mg/day, *p* = 0.029). Traditional demographic and clinical variables, including age, sex, BMI, smoking status, prevalence of hypertension, hemoglobin, magnesium, albumin, immunosuppressive regimen, and NTproBNP, did not differ significantly between groups.

A marked difference was observed in lipid and metabolic profiles. Patients with EDC showed significantly higher triglycerides, TG/HDL ratio, LDL/HDL ratio, TyG index, Atherogenic Index of Plasma (AIP), and a higher prevalence of metabolic syndrome. In contrast, inflammatory markers, including hs-CRP, NLR, CRP/albumin ratio, and CBC-derived indices, did not differ between groups. Left ventricular ejection fraction was preserved in both groups and did not differ significantly between patients with and without EDC (58.8% vs. 60.9%, *p* = 0.16).

### 3.2. Associations Between Metabolic and Inflammatory Markers and Echocardiographic Damage

#### 3.2.1. Univariate Associations with Echocardiographic Damage

In univariate analysis, parameters reflecting insulin resistance and atherogenic dyslipidemia (triglycerides, TG/HDL, LDL/HDL, TyG, AIP, and metabolic syndrome) were associated with EDC, whereas inflammatory markers were not (Table 1). These findings suggested that metabolic phenotype, rather than inflammatory burden, might be more closely linked to cardiac remodeling in this population.

#### 3.2.2. Multivariable Logistic Regression Analysis

Multivariable logistic regression models were constructed to evaluate whether metabolic and inflammatory parameters were independently associated with EDC after adjustment for clinically relevant confounders. Variable selection was based on biological relevance, clinical interpretability, and avoidance of collinearity rather than solely on univariable statistical significance.

Age and sex were included in all models due to their known influence on cardiac structure and metabolic profile. eGFR and proteinuria were included as markers of graft function and endothelial/vascular injury, both of which are known to contribute to cardiac remodeling in KTRs. Metabolic phenotype was primarily assessed using TyG and AIP, which were selected as integrated markers of insulin resistance and atherogenic dyslipidemia, respectively. These indices capture the combined effects of glucose and lipid metabolism and have been shown to provide a more comprehensive assessment of cardiometabolic risk than individual lipid parameters or ratios. Similarly, individual lipid parameters (triglycerides, TG/HDL, LDL/HDL) were not included, together with composite metabolic indices in multivariable models, due to their strong intercorrelation and overlapping biological information. To improve interpretability, TyG was rescaled by a factor of 10, and odds ratios are reported per 0.1-unit increase.

Systemic inflammation was primarily represented by hs-CRP, selected as the main inflammatory covariate due to its established clinical validity and interpretability. Additional inflammatory indices (NLR, NLR/albumin, and CRP/albumin) were evaluated in secondary analyses but were not included simultaneously in primary models to avoid redundancy.

The number of variables included in each model was limited relative to the number of events to reduce the risk of overfitting, ensuring that each model included a limited number of non-redundant variables representing distinct biological pathways.

In models adjusted for these variables, metabolic indices showed strong, consistent independent associations with EDC. TyG remained significantly associated with the presence of echocardiographic damage (OR ≈ 1.13 per 0.1 increase, *p* = 0.001), and this association persisted after further adjustment for hs-CRP and other inflammatory markers (Table 2).

In contrast, inflammatory markers, including hs-CRP, NLR, and CRP/albumin ratio, were not associated with EDC after adjustment. The presence of metabolic syndrome showed only a modest association (OR 2.05, *p* = 0.048), which became non-significant when TyG or AIP were introduced into the models.

These findings indicate that insulin resistance and atherogenic dyslipidemia, rather than systemic inflammation, are the principal determinants of cardiac remodeling in the stable post-transplant phase.

A similar pattern was observed when AIP was evaluated in place of TyG, with AIP showing a strong independent association with EDC (OR = 10.39, 95% CI 2.22–48.71, *p* = 0.003), whereas inflammatory indices remained non-significant.

**Figure 3:**
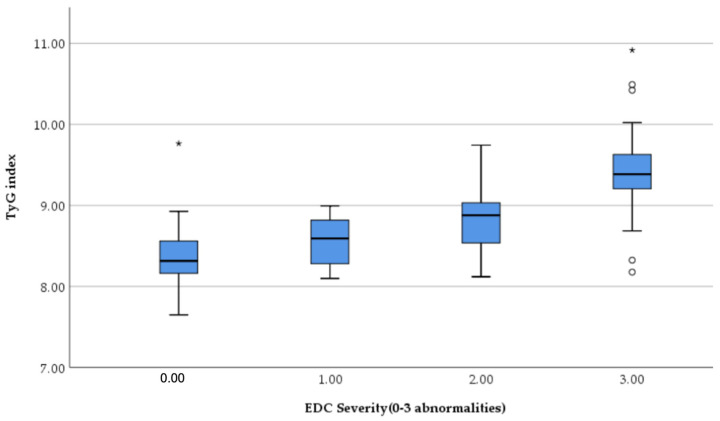
Distribution of TyG index according to echocardiographic damage composite (EDC) severity. TyG values increase progressively with the number of structural and functional cardiac abnormalities (LVH, diastolic dysfunction, left atrial enlargement). A significant dose–response relationship is observed (ANOVA *p* < 0.001). Boxes represent the interquartile range (IQR) with the median indicated by the horizontal line. Circles indicate outliners (1.5–3xIQR) and asterisks * indicate extreme outliners (>3xIQR).

TyG increased progressively with the severity of echocardiographic damage. One-way ANOVA showed a highly significant difference among EDC severity groups (F = 47.3, *p* < 0.001). Post hoc Bonferroni analysis demonstrated that patients with moderate (EDC score 2) and severe remodeling (EDC score 3) had significantly higher TyG values compared with those with no or mild abnormalities, with a clear stepwise increase between each severity level (Figure 3).

**Figure 4:**
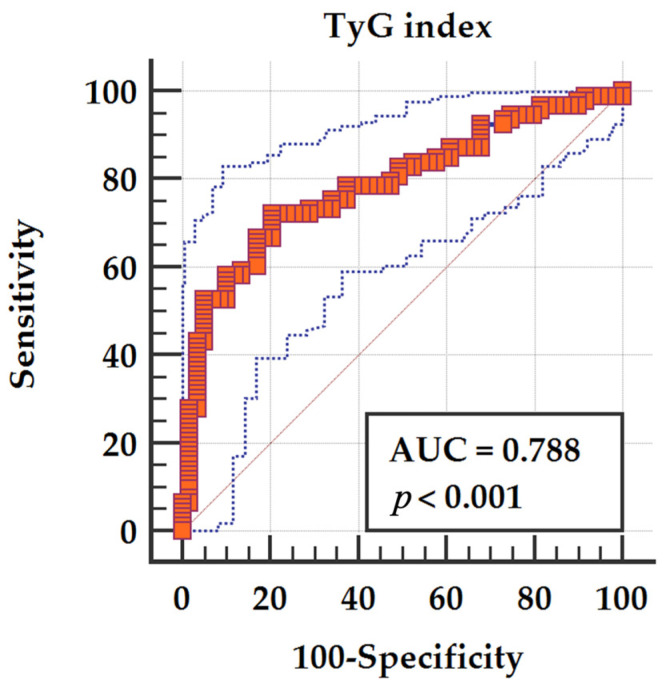
ROC curve showing the ability of the TyG index to discriminate patients with echocardiographic damage.

Receiver operating characteristic (ROC) analysis demonstrated good discriminative ability of TyG for echocardiographic damage, with an AUC of 0.788 (95% CI 0.716–0.860). The optimal cut-off value identified by the Youden index was TyG > 8.78, providing 72.0% sensitivity and 79.7% specificity for the detection of echocardiographic damage (Figure 4).

**Figure 5:**
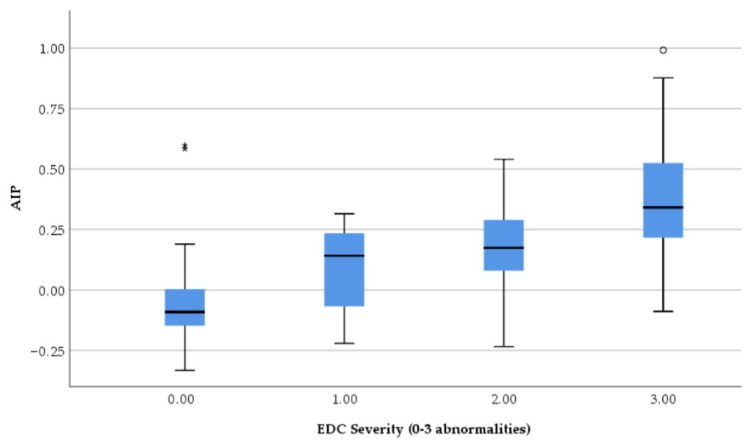
Distribution of AIP according to echocardiographic damage severity. AIP values increase progressively with the number of cardiac abnormalities, demonstrating a significant dose–response relationship (ANOVA *p* < 0.001). Circles indicate outliners and asterisks ** indicate extreme outliners.

AIP also increased progressively with echocardiographic damage severity. One-way ANOVA demonstrated significant differences across EDC groups (F = 29.7, *p* < 0.001). Post hoc Bonferroni analysis showed that patients with moderate and severe remodeling had significantly higher AIP values compared with those with no or mild abnormalities, confirming a clear dose–response relationship (Figure 5).

**Figure 6:**
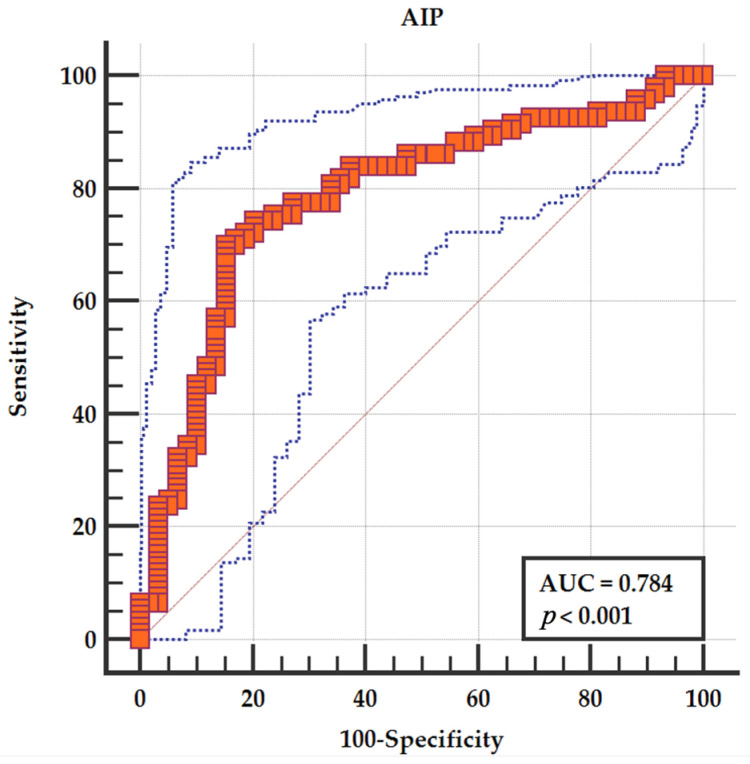
ROC curve showing the ability of AIP to discriminate patients with echocardiographic damage.

ROC analysis showed that the Atherogenic Index of Plasma (AIP) also demonstrated good discriminative ability for echocardiographic damage (AUC 0.784, 95% CI 0.710–0.856, *p* < 0.001). The optimal cut-off value identified by the Youden index was AIP > 0.144, yielding a sensitivity of 70% and a specificity of 84.75% (Figure 6).

These analyses demonstrate that markers of insulin resistance and atherogenic dyslipidemia (TyG and AIP) are independently associated with echocardiographic cardiac remodeling in KTRs, whereas metabolic syndrome has a borderline association, and inflammatory markers do not show independent associations after adjustment for graft function and demographic factors.

### 3.3. Clinical Outcomes During Follow-Up

From the baseline evaluation (12 ± 4 months post-transplant) to the end of follow-up, December 2025, 78 patients experienced a Major Adverse Clinical Outcome (MACO), defined as the first occurrence of cardiovascular event, graft failure requiring return to dialysis, or death. A cardiovascular-specific composite outcome (MACCE) occurred in 49 patients.

This high event rate allowed evaluation of whether the baseline cardiometabolic phenotype and echocardiographic damage were associated with subsequent clinical evolution. Because predictors were measured at a standardized baseline evaluation (~12 months post-transplant), follow-up was calculated from this time point. Transplant vintage (10.25 ± 4.87 years) at final evaluation reflects follow-up duration rather than baseline exposure and was therefore not analyzed as a predictor.

#### 3.3.1. Baseline Characteristics According to MACO

Patients who developed MACO were older and had markedly worse graft function, higher proteinuria, lower hemoglobin and albumin levels, and substantially higher NT-proBNP. In contrast to the echocardiographic cross-sectional analysis, inflammatory markers (hs-CRP, NLR, CRP/albumin, PLR) were significantly higher in patients who later developed MACO (Table 3). Metabolic indices were also significantly different, with higher TG/HDL and LDL/HDL ratios, higher TyG and AIP, and a higher prevalence of metabolic syndrome in the MACO group.

Furthermore, echocardiographic damage composite (EDC) at baseline was more frequent in patients with MACO, although this difference did not reach statistical significance. Notably, while inflammatory markers were not significantly associated with EDC at baseline, they showed a different pattern in longitudinal analyses, being associated with overall adverse clinical outcomes (MACO). This suggests that metabolic abnormalities are more closely related to structural cardiac remodeling, whereas systemic inflammation may reflect processes driving subsequent clinical deterioration. The lack of association between inflammatory markers and EDC, contrasted with their association with overall adverse clinical outcomes, likely reflects differences in underlying pathophysiological mechanisms. Echocardiographic remodeling primarily reflects chronic myocardial adaptation driven by metabolic and hemodynamic factors, including insulin resistance and dyslipidemia, which promote hypertrophy and fibrosis. In contrast, systemic inflammation represents a broader biological process reflecting immune activation, endothelial dysfunction, and multi-organ stress. As such, inflammatory markers may be more closely related to overall clinical vulnerability, including graft dysfunction, infection, and mortality, rather than to localized structural cardiac changes.

#### 3.3.2. Baseline Characteristics According to MACCE

Patients who experienced MACCE showed a similar pattern of older age, worse graft function, higher proteinuria, lower albumin, and higher NTproBNP. Metabolic parameters were strongly associated with MACCE, particularly TyG, AIP, TG/HDL, LDL/HDL, and metabolic syndrome. In contrast to MACO, inflammatory markers were less consistently associated with MACCE (Table 4).

These observations suggested that the inflammatory burden may be more closely related to global adverse outcomes, while metabolic phenotype may be more specifically linked to cardiovascular events.

#### 3.3.3. Multivariable Time-to-Event Analysis

Multivariable Cox proportional hazards models were constructed to determine whether baseline metabolic phenotype, inflammatory status, or echocardiographic damage independently predicted clinical outcomes during follow-up. Variable selection was based on biological relevance, clinical interpretability, and avoidance of collinearity rather than solely on univariable significance.

Age and sex were included in all models due to their established influence on cardiovascular risk. eGFR and proteinuria were systematically included as markers of graft function and endothelial injury, both of which are strongly associated with outcomes in transplant populations. Metabolic phenotype was primarily assessed using TyG and AIP as integrated markers of insulin resistance and atherogenic dyslipidemia. Because metabolic indices (TyG, AIP, metabolic syndrome, TG/HDL, and LDL/HDL) represent overlapping biological pathways, they were not included simultaneously in the same model and were tested in separate models. Inflammatory markers were then introduced to evaluate whether metabolic parameters retained predictive value beyond systemic inflammation. Systemic inflammation was primarily represented by hs-CRP, chosen as the main inflammatory covariate due to its established clinical validity and widespread use as a marker of systemic inflammatory burden. Additional inflammatory indices were explored in secondary analyses but were not included in the primary models simultaneously to avoid redundancy.

In the base model, including age, sex, eGFR, and proteinuria, graft dysfunction emerged as the dominant independent predictor of MACO. When inflammatory markers were added to the model, they showed a borderline significant association after adjustment. Metabolic indices such as TyG were not associated with MACO in these models (Table 5).

When NLR was used instead of CRP in the multivariable Cox model for MACO, both NLR (HR 1.204, *p* < 0.001) and TyG (HR 1.051 per 0.1 increase, *p* = 0.025) showed significant associations, suggesting partial overlap between inflammatory and metabolic pathways captured by this marker. EDC did not independently predict MACO after adjustment for graft function and inflammation (HR = 0.837, 95% CI 0.329–2.131, *p* = 0.709). These findings indicate that systemic inflammation and graft dysfunction are the dominant determinants of overall adverse clinical trajectory after transplantation.

When MACCE was used as the outcome, a different pattern emerged. In Cox proportional hazards models adjusted for age, sex, eGFR, and proteinuria, TyG remained independently associated with the hazard of cardiovascular events during follow-up. This association persisted after additional adjustment for inflammatory markers, which did not show independent significance (Table 6).

Similar results were observed when AIP was tested in place of TyG (HR = 10.8, 95% CI 3.06–38.11 *p* < 0.001), confirming the role of atherogenic dyslipidemia in cardiovascular risk.

EDC did not independently predict MACCE when metabolic indices were included in the model (HR = 0.967, 95% CI 0.506–1.847, *p* = 0.919).

In summary, graft dysfunction and inflammation predict overall adverse outcome (MACO). Metabolic phenotype (TyG/AIP) specifically predicts cardiovascular events (MACCE). Baseline echocardiographic damage reflects metabolic remodeling but does not independently predict outcomes once graft function and metabolic indices are considered.

#### 3.3.4. Kaplan–Meier Analysis

**Figure 7:**
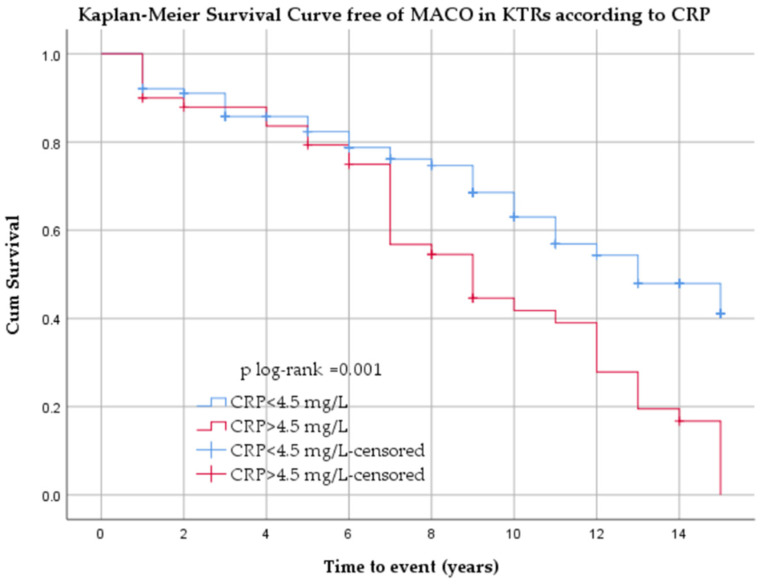
Kaplan–Meier curves for MACO according to CRP category.

Kaplan–Meier analysis showed significantly lower MACO-free survival in patients with CRP above the median value (>4.5 mg/L) at baseline compared with those with lower values (log-rank *p* = 0.001) (Figure 7).

**Figure 8:**
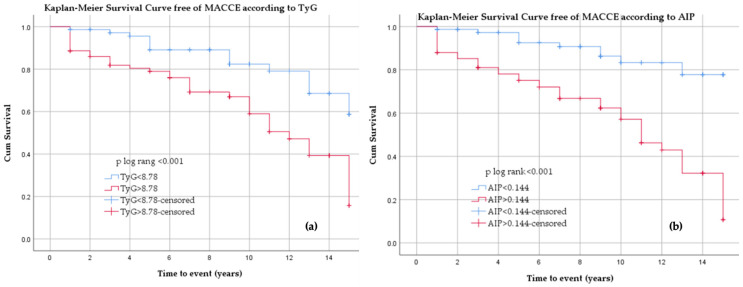
Kaplan–Meier curves for MACCE according to TyG (**a**) and AIP (**b**) categories.

Kaplan–Meier analysis demonstrated a markedly higher incidence of MACCE in patients with TyG values above the ROC-derived cut-off (>8.78) compared with those with lower values. Event-free survival diverged early after baseline and continued to separate over time (log-rank *p* < 0.001) (Figure 8a).

Kaplan–Meier analysis demonstrated significantly lower MACCE-free survival in patients with AIP values above the ROC-derived cut-off (>0.144) compared with those with lower values at baseline (log-rank *p* < 0.001) (Figure 8b).

## 4. Discussion

This study evaluated the relationships among metabolic phenotype, inflammatory status, and cardiovascular involvement in KTRs at a stable post-transplant time point and during subsequent follow-up. Two complementary observations emerged.

First, at approximately one year after transplantation, echocardiographic cardiac remodeling, defined by the presence of left ventricular hypertrophy, diastolic dysfunction, or left atrial enlargement, was strongly associated with markers of insulin resistance and atherogenic dyslipidemia, particularly the TyG index and the AIP. In contrast, classical inflammatory markers derived from hs-CRP, CBC indices, and their albumin ratios showed no association with echocardiographic damage. Importantly, the association between TyG and cardiac remodeling remained robust after adjustment for age, sex, graft function, proteinuria, and inflammatory markers. The choice of a baseline evaluation approximately 1 year after transplantation enabled assessment of a stable post-transplant cardiometabolic state, thereby avoiding the confounding effects of early peri-transplant inflammation and hemodynamic changes.

Second, during follow-up, the determinants of adverse clinical outcomes varied by endpoint type. Systemic inflammation and graft dysfunction were the dominant predictors of the composite adverse clinical outcome (MACO), whereas metabolic phenotype remained specifically associated with MACCE. The differential associations observed in this study likely reflect distinct underlying pathophysiological pathways. Insulin resistance and atherogenic dyslipidemia, as captured by TyG and AIP, are known to promote myocardial remodeling through mechanisms including altered myocardial substrate utilization, lipotoxicity, oxidative stress, and interstitial fibrosis. These processes primarily affect cardiac structure and diastolic function, leading to left ventricular hypertrophy and increased filling pressures, even in the presence of preserved ejection fraction, a pattern characteristic of heart failure with preserved ejection fraction [55,56]. However, these metabolic alterations appear to exert a more organ-specific effect, predominantly influencing myocardial structure and the development of cardiovascular events, rather than the broader spectrum of adverse outcomes captured by MACO. In contrast, MACO represents a heterogeneous composite outcome that includes not only cardiovascular events but also graft failure and mortality, which are influenced by multiple additional factors such as graft dysfunction, infections, and systemic comorbidities.

In contrast, systemic inflammation represents a broader and less specific biological process, reflecting immune activation, endothelial dysfunction, and ongoing injury at multiple organ levels, including the kidney graft. In CKD and transplant populations, inflammatory markers have been consistently associated with adverse outcomes, including mortality and graft dysfunction, rather than with localized structural cardiac changes [57]. This mechanistic divergence may explain why metabolic indices are strongly associated with subclinical cardiac remodeling and cardiovascular events, whereas inflammatory markers are more consistently associated with global adverse outcomes (MACO) in kidney transplant recipients. Importantly, these pathways are not mutually exclusive but may act in parallel, with metabolic abnormalities driving structural cardiac changes and inflammation reflecting systemic vulnerability that determines long-term clinical outcomes.

Together, these findings outline a pathophysiological sequence in which metabolic abnormalities shape early cardiac remodeling, while inflammation and graft dysfunction govern global clinical deterioration, and metabolic phenotype continues to drive cardiovascular risk.

### 4.1. Cardiac Remodeling Phenotype After Kidney Transplantation

The EDC used in this study (LV hypertrophy, diastolic dysfunction, and left atrial enlargement) represents the typical cardiac phenotype observed after kidney transplantation. This pattern reflects chronic pressure and volume overload, increased ventricular stiffness, and long-standing elevation of filling pressure rather than systolic dysfunction. Such remodeling is highly prevalent in transplant populations and is known to be associated with adverse cardiovascular outcomes. Previous echocardiographic studies in KTRs have reported heterogeneous patterns of cardiac remodeling after transplantation. While some investigators observed regression of left ventricular hypertrophy with parallel improvement in systolic function and a reduction in the severity of mitral and tricuspid regurgitation [58], others documented persistent diastolic dysfunction, elevated pulmonary pressures, and pericardial abnormalities, particularly in patients with impaired graft function, similar to associated HD findings [6,59]. Furthermore, regression of left ventricular hypertrophy is not always associated with an improvement of ejection fraction, and severe pre-transplant diastolic dysfunction and reduced ejection fraction have been identified as predictors of post-transplant mortality, underscoring the prognostic relevance of residual cardiac remodeling [60].

Our findings are consistent with this body of evidence. Despite systematic cardiovascular screening before transplantation and predominantly preserved systolic function, more than half of the recipients in our cohort exhibited residual diastolic remodeling one year after transplantation. This supports the concept that CKD-related cardiac structural and functional alterations frequently persist after Tx and may not fully regress, even in the absence of overt pre-existing cardiovascular disease. In our study, this residual remodeling was closely associated with metabolic abnormalities rather than inflammatory markers, suggesting that post-transplant cardiometabolic factors may contribute to the persistence or progression of diastolic cardiac phenotype after transplantation.

Although metabolic syndrome was more frequent among patients with echocardiographic damage and showed a modest association in multivariable analysis, its effect was substantially attenuated when TyG or AIP were introduced into the models. This suggests that composite definitions such as metabolic syndrome may be insufficiently sensitive to capture the specific metabolic disturbances driving myocardial remodeling in transplant recipients.

In contrast, TyG and AIP continuous indices reflecting insulin resistance and atherogenic dyslipidemia showed strong, consistent, and independent associations. These markers appear to better represent the cardiometabolic processes contributing to myocardial structural changes in this population.

A particularly relevant finding is the absence of association between inflammatory markers and echocardiographic remodeling. Although chronic inflammation is a central driver of cardiovascular injury in chronic kidney disease, this relationship may attenuate after transplantation. Immunosuppressive therapy, correction of the uremic milieu, and removal of dialysis-related inflammatory stimuli may reduce the impact of systemic inflammation on cardiac structure. In this stable post-transplant phase, metabolic and lipid-related pathways may become the predominant determinants of myocardial remodeling.

### 4.2. From Remodeling to Outcomes: Divergence of Metabolic and Inflammatory Pathways

During follow-up, a different pattern emerged. When the overall adverse clinical outcome (MACO) was considered, inflammatory markers and graft dysfunction were strongly associated with events, whereas TyG lost significance after adjustment. This indicates that systemic inflammation becomes a key determinant of global clinical deterioration, including graft loss and mortality.

In contrast, when cardiovascular events alone (MACCE) were analyzed, TyG remained a consistent and independent predictor even after adjustment for inflammatory markers, which were not significant. This demonstrates that the metabolic phenotype continues to play a specific role in cardiovascular risk, whereas chronic inflammation contributes more broadly to the overall adverse trajectory, as in several pathological conditions in HD [61,62].

Interestingly, when NLR was used instead of CRP in multivariable models, both NLR and TyG remained significant predictors of MACO. This may reflect that NLR, unlike CRP, captures not only systemic inflammation but also elements of metabolic stress and endothelial dysfunction, partially overlapping with the metabolic pathway reflected by TyG. In contrast, for MACCE, NLR lost significance, further supporting the concept that cardiovascular events in this cohort are primarily driven by metabolic rather than inflammatory mechanisms. The inclusion of additional inflammatory biomarkers would have enhanced the scientific value of our study; however, because of its retrospective design, these data were unavailable.

This divergence is physiologically plausible. Insulin resistance and atherogenic dyslipidemia promote myocardial remodeling and atherosclerotic burden, predisposing to cardiovascular events. In contrast, inflammation and graft dysfunction reflect systemic deterioration that influences survival and graft outcome.

### 4.3. Clinical and Pathophysiological Implications

KTRs face a markedly elevated risk of cardiovascular events compared with the general population, a burden partly driven by insulin resistance. The TyG index (a surrogate marker of insulin resistance) has been linked to the emergence of coronary artery disease and stroke, affecting both high-risk individuals and those without a history of atherosclerosis [63].

Furthermore, the TyG index can predict CKD onset in the general population, and higher TyG values are associated with a faster progression toward end-stage kidney disease [63,64]. A significant association between the TyG index and MACE occurrence was demonstrated in the NEFRONA observational study, which included 1142 non-diabetic patients with chronic kidney disease [29]. The crucial role of insulin resistance in linking CKD to cardiovascular disease was also underscored by a significant cross-sectional analysis using data from the National Health and Nutrition Examination Survey (NHANES) [65].

Similar results to those presented for our cohort were observed in other studies. In a retrospective study of non-diabetic KTRs, the TyG index was identified as an independent predictor of MACCEs [66]. The analysis of a cohort of 715 consecutive KTRs monitored over nearly a decade demonstrated that a higher TyG index correlated with an increased incidence of cardiovascular events. Alarmingly, up to 80% of these patients exhibited a TyG value exceeding 4.49, a number suggested as a benchmark for insulin resistance in the general population [67], highlighting the extensive metabolic issues within this group [68]. Similarly, AIP has emerged as a robust marker of plasma atherogenicity and cardiovascular risk, reflecting the balance between protective and atherogenic lipoproteins and the presence of small, dense LDL particles. Given the intricate nature of lipid metabolism, AIP may provide a more precise representation of plasma atherogenicity than individual lipid measurements [69]. Elevated triglyceride levels contribute to the creation of small, dense LDL cholesterol particles, which are more prone to oxidative modification, thus accelerating atherosclerosis [70,71]. Oxidized LDL is taken up by macrophages, leading to foam cell formation and accumulation in arterial walls, which facilitates the development and progression of atherosclerotic plaques. As these plaques grow and become unstable, they narrow the lumen and hinder blood flow, raising the risk of heart attacks, strokes, and cardiovascular-related deaths. Additionally, insulin resistance, often linked to high AIP levels and prevalent in chronic kidney disease patients, intensifies this process. Elevated AIP levels may interfere with insulin signaling pathways, reduce cellular insulin sensitivity, and promote insulin resistance [72,73].

Previous research has highlighted a significant link between the AIP and cardiovascular disease within the general population [74,75,76]. A cohort study using data from the National Health and Nutrition Examination Survey (NHANES), spanning 1999 to 2018, included 4403 participants and found a significant independent association between the AIP and both all-cause and specific mortality with a median follow-up of 83 months. Notably, a linear dose–response relationship was identified, indicating that higher AIP levels correlate with poorer outcomes in patients with chronic kidney disease [77]. Interestingly, studies in dialysis populations have reported a U-shaped association between AIP and mortality, with both low and high values associated with adverse outcomes, partly reflecting the coexistence of malnutrition–inflammation and atherogenic dyslipidemia in end-stage kidney disease [78]. In contrast, in the post-transplant setting where severe malnutrition is less prevalent, our findings show a direct association between higher AIP and cardiovascular risk, consistent with a predominantly metabolic mechanism. In a study utilizing data from the China Health and Retirement Longitudinal Study (CHARLS), researchers examined participants diagnosed with cardiovascular–kidney–metabolic (CKM) syndrome to explore the relationship between AIP and cardiovascular disease (CVD). Elevated cumulative AIP values and suboptimal long-term AIP control were associated with a higher incidence of CVD, especially among individuals in CKM syndrome stages 1–3 [79].

Our findings extend these observations to the transplant population and, importantly, demonstrate how these indices relate not only to clinical cardiovascular events but also to structural cardiac remodeling. The parallel behavior of TyG and AIP with respect to both echocardiographic damage and MACCE strongly supports the role of insulin resistance and atherogenic dyslipidemia as central mechanisms underlying post-transplant cardiovascular involvement.

### 4.4. Role of Graft Function and Endothelial Injury

Lower eGFR and higher proteinuria were consistently associated with both echocardiographic damage and adverse outcomes. These parameters likely represent endothelial injury and vascular dysfunction, which act synergistically with metabolic abnormalities in shaping cardiovascular risk in transplant recipients. Importantly, the associations of TyG and AIP with both echocardiographic damage and cardiovascular events remained independent of graft function, underscoring the specific contribution of the metabolic phenotype.

### 4.5. Emerging Therapies for Cardiometabolic Remodeling After Kidney Transplantation

The strong association between metabolic phenotype and cardiac remodeling observed in this study has potential therapeutic implications. Interventions targeting insulin resistance and atherogenic dyslipidemia may represent promising strategies to attenuate residual diastolic remodeling after kidney transplantation.

SGLT2 inhibitors have demonstrated cardiorenal protective effects in the general population through multiple direct and pleiotropic mechanisms, including improved glycemic control, amelioration of anemia, optimization of volume status, reduction in glomerular hyperfiltration, modulation of intestinal pathways, and improved blood pressure control. Additionally, they decrease cardiac preload and afterload and exert anti-inflammatory effects at the endothelial level by reducing oxidative stress and proinflammatory cytokine expression [80,81]. In KTRs, given their exclusion from the pivotal registration trials such Dapagliflozin Effect on Cardiovascular Events—Thrombolysis in Myocardial Infarction 58 (DECLARE–TIMI 58), Dapagliflozin And Prevention of Adverse Outcomes in Chronic Kidney Disease (DAPA-CKD), Empagliflozin Outcome Trial in Patients With Chronic Heart Failure and a Reduced Ejection Fraction (EMPEROR-Reduced), and The Study of Heart and Kidney Protection With Empagliflozin (EMPA-KIDNEY), their utility was subsequently explored in real-world settings, often beyond the scope of approved label indications. The main concerns regarding their use were the potential increase in the risk of urinary tract infection and the possibility of pharmacological interactions with immunosuppressive therapy. The use of SGLT2 inhibitors in non-diabetic KTRs remains controversial, though some meta-analyses support benefits, including for calcineurin inhibitor–related hyperuricemia and hypomagnesemia [82]. Studies have also demonstrated reductions in body weight and a slower decline in renal graft function, with no significant increase in adverse events [83]. Another meta-analysis did not demonstrate a significant effect in preventing the decline of renal graft function [84]. In KTRs with diabetes mellitus, the efficacy of SGLT2 inhibitors has been confirmed for glycemic control and for their renoprotective and cardioprotective effects. However, although there is sufficient evidence to support the use of SGLT2 inhibitors in KTRs with diabetes mellitus, the available data remain insufficient to justify their use in non-diabetic transplant recipients [85].

In the general population, glucagon-like peptide-1 receptor agonists (GLP-1 RAs) have demonstrated beneficial effects on heart failure, reducing symptoms through weight loss and improving outcomes, particularly in heart failure with preserved ejection fraction. Studies of tirzepatide and semaglutide have shown favorable cardiovascular effects in patients with dysmetabolic syndrome; however, their impact on hard clinical outcomes, including cardiovascular mortality and hospitalizations, has not yet been fully established [86]. A recent meta-analysis of 7000 KTRs found that both SGLT2 inhibitors and GLP-1 receptor agonists improved overall survival, cardiovascular outcomes, and renal prognosis. The analysis also found that SGLT2 inhibitor therapy was associated with improvements in serum magnesium and uric acid levels [82].

Non-steroidal mineralocorticoid receptor antagonists (MRAs) with a lower adverse-effect profile than steroidal aldosterone antagonists, including a reduced risk of hyperkalemia, have been studied in KTRs only minimally. The only study to include organ transplant patients, the EFFECTOR trial, is currently ongoing. The potential benefits of these agents are multisystemic, reflecting the widespread distribution of mineralocorticoid receptors in endothelial cells, vascular smooth muscle, renal tubular cells, podocytes, mesangial cells, and remodeling-associated cells such as fibroblasts. Receptor blockade mediates multiple protective effects, including mitigation of ischemia–reperfusion injury in both renal and cardiac tissues, reduction in inflammation and fibrosis (with consequent reduction in proteinuria), and counteraction of the hemodynamic effects induced by calcineurin inhibitors [87].

Dyslipidemia is usually treated with statins, fibrates, or ezetimibe, but efficacy is limited under immunosuppression, especially with mTOR inhibitors, while PCSK9 inhibitors are still under study. This highlights the gap between the need for aggressive cardiovascular prevention and the lack of coordinated, evidence-based strategies.

This study provides a novel perspective by evaluating cardiometabolic and inflammatory phenotypes at a physiologically stable time point after Tx and by integrating structural cardiac assessment with longitudinal clinical outcomes. It demonstrates that metabolic and inflammatory pathways contribute differently at distinct stages of cardiovascular involvement after kidney transplantation. 

### 4.6. Applicability in Clinical Practice

The present findings have important clinical applicability. Simple metabolic indices such as TyG and AIP, which are easily derived from routine laboratory parameters, may serve as practical tools for the early identification of KTRs at increased risk of subclinical cardiac remodeling and subsequent cardiovascular events. Their use could help refine cardiovascular risk stratification beyond traditional risk factors and inflammatory markers.

Patients with elevated TyG or AIP values may benefit from closer cardiovascular surveillance, including more detailed echocardiographic evaluation and earlier detection of diastolic dysfunction or structural abnormalities. In addition, these findings support the potential value of targeting metabolic abnormalities after Tx through optimization of glycemic control, lipid management, lifestyle interventions, and the use of emerging cardiometabolic therapies.

Conversely, the association between inflammatory markers and overall adverse outcomes suggests that systemic inflammation may identify patients at risk of global clinical deterioration, including graft dysfunction and mortality, and may warrant closer monitoring of graft function and comorbid conditions. Although systemic inflammation is also potentially modifiable in this population, it reflects a complex interplay of graft function, immune activation, infections, and comorbidities, and is therefore less directly targetable than metabolic abnormalities. Its management relies on optimization of graft function, careful adjustment of immunosuppressive therapy, and treatment of intercurrent conditions rather than on a single targeted intervention.

#### Limitations

This study has several limitations that should be acknowledged. First, its retrospective design limits the ability to establish causal relationships and may introduce selection bias. Second, the study was conducted in a single center, which may limit the generalizability of the findings to other transplant populations. Third, the relatively small sample size and number of events may reduce statistical power, and causal inferences cannot be drawn from the observed associations between echocardiographic, metabolic, and inflammatory parameters and MACO/MACCE.

Despite adjustment for clinically relevant confounders, residual confounding cannot be excluded, particularly given the complex interplay between metabolic, inflammatory, and transplant-related factors. Echocardiographic, metabolic, and inflammatory parameters were assessed at a single time point, and their temporal variability during follow-up was not captured. Similarly, graft function was evaluated only at baseline, and longitudinal changes in eGFR were not assessed. Given the dynamic nature of graft function, the trajectory of eGFR decline may provide additional prognostic information and should be explored in future studies. uACR, an important marker of kidney injury and disease progression, was not available for all of our patients; therefore, it was not included in the statistical analysis. The decline in renal function over the course of graft survival was not assessed due to insufficient laboratory data. The use of ACE inhibitors and ARBs, known to modulate the immune response, was not reported, as data on antihypertensive therapy class and dosage were insufficient.

Although echocardiographic assessment was performed according to current recommendations, the composite definition of cardiac remodeling may not fully capture all aspects of myocardial dysfunction, particularly subtle alterations in systolic function or myocardial strain. In addition, inflammatory status was assessed using systemic markers, which may not fully reflect local myocardial inflammatory processes.

Overall, this study provides insight into the associations between echocardiographic findings, metabolic and inflammatory markers, and post-transplant cardiovascular outcomes; however, these findings should be considered hypothesis-generating. Future studies with larger, multicenter cohorts and longitudinal assessment of metabolic and inflammatory profiles are warranted to confirm and extend these results.

## 5. Conclusions

In kidney transplant recipients, insulin resistance and atherogenic dyslipidemia are strongly associated with cardiac remodeling one year after transplantation and remain specifically linked to subsequent cardiovascular events. TG and AIP may be useful for cardiovascular risk stratification in renal transplant recipients and for the early detection of subclinical cardiac conditions that may progress post-transplant. The post-transplant inflammatory syndrome has a multifactorial etiology, including renal graft dysfunction, immunosuppressive levels, systemic inflammation, and infectious status. Together, these findings support the concept that metabolic and inflammatory pathways play distinct yet complementary roles: metabolic abnormalities represent more specific and potentially modifiable drivers of cardiac remodeling, whereas inflammation reflects a broader state of clinical vulnerability associated with adverse outcomes.

## Figures and Tables

**Table 1:** Baseline characteristics of the study group.

Parameters	All 152	EDC (78)	No EDC (74)	*p*
Sex M	81/152 (53.29%)	45/78 (57.69%)	36 (48.65%)	0.36
Age (years)	50.71 ± 11.86	52.40 ± 10.83	48.75 ± 12.74	0.06
BMI (kg/m^2^)	24.17 ± 3.91	24.75 ± 3.59	23.54 ± 4.20	0.058
Smoking (yes)	28/152 (18.66%)	16/78 (20.51%)	12/74 (16.22%)	0.52
eGFR (mL/min/1.73)	45.41 ± 25.25	40.72 ± 23.52	50.02 ± 26.23	0.02
hs-CRP (mg/L)	11.44 ± 19.12	11.32 ± 13.95	11.35 ± 23.63	0.99
Hb (g/dL)	12.12 ± 2.21	12.01 ± 2.30	12.20 ± 2.13	0.59
Mg (mg/dL)	1.71 ± 0.25	1.71 ± 0.24	1.69 ± 0.25	0.60
Albumin (g/dL)	39.38 ± 5.11	39.77 ± 4.81	38.96 ± 5.45	0.33
Proteinuria (mg/24 h)	902.66 ± 1280.35	1127.68 ± 1513.22	672.95 ± 933.94	0.029
Glycemia (mg/dL)	97.70 ± 26.25	100.78 ± 27.34	94.56 ± 24.96	0.147
Tacrolimus	104/152 (68.42%)	52/78 (66.66%)	52/74 (70.27%)	0.75
CsA	39/152 (25.66%)	22/78 (28.21%)	17/74 (22.97%)	0.46
Rap	8/152 (5.26%)	3/78 (3.85%)	5/74 (6.76%)	0.42
Neo	26/152 (17.11%)	13/78 (16.66%)	13/74 (17.57%)	0.85
Hypertension	148/152 (97.375)	77/78 (98.72%)	70/74 (94.59%)	0.28
NTproBNP (pg/mL)	5326.32 ± 8926.94	5117.433 ± 7175.14	5694.94 ± 11,643.49	0.83
HDL cholesterol (mg/dL)	45.91 ± 12.07	44.23 ± 11.88	47.62 ± 12.16	0.086
TG (mg/dL)	161.39 ± 89.12	187.79 ± 107.80	132.93 ± 51.47	<0.001
TG/HDL cholesterol	3.96 ± 2.98	4.69 ± 3.33	3.20 ± 2.37	0.002
LDL/HDL cholesterol	2.87 ± 1.42	3.13 ± 1.23	2.59 ± 1.57	0.021
TyG index	8.84 ± 0.55	9.02 ± 0.57	8.65 ± 0.47	<0.001
AIP	0.16 ± 0.25	0.24 ± 0.25	0.07 ± 0.23	<0.001
PLR	174.45 ± 101	180.27 ± 100	170.18 ± 110	0.78
NLR/albumin	0.09 ± 0.06	0.097 ± 0.067	0.088 ± 0.045	0.33
CRP/albumin	0.31 ± 0.52	0.302 ± 0.390	0.305 ± 0.637	0.97
NLR	3.55 ± 2.11	3.77 ± 2.49	3.31 ± 1.60	0.18
MetS	62/152 (40.79%)	41/78 (52.56%)	21/74 (28.38%)	0.03
LVEF	59.84 ± 10.09	58.8 ± 10.15	60.9 ± 8.67	0.16

Legend: BMI—body mass index, eGFR—estimated glomerular filtration rate, hs-CRP—high sensitivity C reactive protein, Hb—hemoglobin, Mg—magnesium, CsA—ciclosporin A, Rap—rapamycin, Neo—neoplastic diseases, NTproBNP—N-terminal pro-B-type natriuretic peptide, HDL cholesterol—high density lipoprotein cholesterol, TG—triglycerides, LDL cholesterol—low density lipoprotein cholesterol, TyG—triglyceride glucose index, AIP—atherogenic index of plasma, PLR—platelet to lymphocyte ratio, NLR—neutrophils to lymphocyte ratio, MetS—metabolic syndrome, LVEF—left ventricular ejection fraction.

**Table 2:** Multiple regression analysis for EDC predictors.

Parameters	OR	95% CI	*p*
TyG_01	1.130	1.051–1.215	0.001
eGFR	0.992	0.975–1.009	0.368
Proteinuria	1.000	1.000–1.001	0.329
Age	1.024	0.993–1.057	0.130
Sex	0.789	0.387–1.609	0.514
hs-CRP	0.991	0.970–1.013	0.434

Legend: TyG—triglyceride glucose index, eGFR—glomerular filtration rate, hs-CRP—high specific C-reactive protein.

**Table 3:** Baseline characteristics of the study cohort according to MACO.

Parameters	All 152	MACO (78)	No MACO (74)	*p*
Sex M	81/152 (53.29%)	39/78 (50%)	42/74 (56.75%)	0.42
Age (years)	50.71 ± 11.86	52.77 ± 11.59	48.55 ± 11.84	0.029
BMI (kg/m^2^)	24.17 ± 3.91	23.87 ± 4.04	24.48 ± 3.78	0.341
Smoking (yes)	28/152 (18.66%)	17/78 (21.79%)	11/74 (14.86%)	0.301
eGFR (mL/min/1.73 m^2^)	45.41 ± 25.25	33.40 ± 19.85	58.08 ± 24.21	<0.001
hs-CRP (mg/L)	11.44 ± 19.12	18.45 ± 24.67	4.15 ± 3.47	<0.001
Hb (mg/dL)	12.12 ± 2.21	11.08 ± 2.24	13.20 ± 1.56	<0.001
Mg (mg/dL)	1.71 ± 0.25	1.70 ± 0.26	1.70 ± 0.23	0.89
Albumin (g/dL)	39.38 ± 5.11	37.51 ± 5.32	41.34 ± 4.07	<0.001
Proteinuria (mg/24 h)	902.66 ± 1280.35	1474.74 ± 1491.32	299.656 ± 571.57	<0.001
Glycemia (mg/dL)	97.70 ± 26.25	99.95 ± 30.64	95.32 ± 20.58	0.27
Tacrolimus	104/152 (68.42%)	46/78 (58.97%)	58/74 (78.38%)	0.01
CsA	39/152 (25.66%)	23/78 (29.49%)	16/74 (21.62%)	0.26
RAP	8/152 (5.26%)	8/78 (10.26%)	0/74 (0%)	0.007
Neo	26/152 (17.11%)	17/78 (21.79%)	9/74 (12.16%)	0.13
Hypertension	148/152 (97.375)	78/78 (100%)	70/74 (94.59%)	0.28
Beta blockers	132/152 (86.84%)	73/78 (93.59%)	59/74 (79.73%)	0.01
Statins	91/152 (59.87%)	48/78 (61.54%)	43/78 (55.13%)	0.74
NTproBNP (pg/mL)	5326.32 ± 8926.94	9975.636 ± 11,256.57	1234.92 ± 2148.52	0.002
HDL cholesterol (mg/dL)	45.91 ± 12.07	42.68 ± 12.62	49.31 ± 10.51	0.001
TG (mg/dL)	161.39 ± 89.12	175.51 ± 71.51	146.51 ± 102.95	0.045
TG/HDL cholesterol	3.96 ± 2.98	4.59 ± 2.69	3.31 ± 3.15	0.008
LDL/HDL cholesterol	2.87 ± 1.42	3.31 ± 1.64	2.39 ± 0.96	<0.001
TyG	8.84 ± 0.55	8.96 ± 0.56	8.71 ± 0.52	0.005
AIP	0.16 ± 0.25	0.24 ± 0.24	0.07 ± 0.24	<0.001
PLR	174.45 ± 101	191.36 ± 97	155.29 ± 103	0.029
NLR/alb	0.09 ± 0.06	0.11 ± 0.07	0.07 ± 0.031	<0.001
CRP/alb	0.31 ± 0.52	0.50 ± 0.67	0.103 ± 0.092	<0.001
NLR	3.55 ± 2.11	4.18 ± 2.57	2.91 ± 1.20	<0.001
MetS	62/152 (40.79%)	41/78 (52.56%)	21/74 (28.38%)	0.03
EDC	78/152 (51.32%)	44/78 (56.41%)	34/74 (45.95%)	0.19

Legend: BMI—body mass index, eGFR—glomerular filtration rate, Hb—hemoglobin, Mg—magnesium, CsA—ciclosporin A, RAP–rapamycin, Neo—neoplastic disorders, NTproBNP—N terminal pro-B-type natriuretic peptide, HDL cholesterol—high density lipoprotein cholesterol, TG—triglycerides, LDL cholesterol—low density lipoprotein cholesterol, TyG—triglyceride glucose index, AIP—atherogenic index of plasma, PLR—platelet to lymphocyte ratio, NLR—neutrophils to lymphocyte ratio, hs-CRP—C reactive protein, MetS—metabolic syndrome, EDC—echocardiographic damage composite.

**Table 4:** Baseline characteristics of study cohort according to MACCE.

Parameters	All 152	MACCE (49)	No MACCE (103)	*p*
Sex M	81/152 (53.29%)	25/49 (50%)	56/103 (56.75%)	0.73
Age (years)	50.71 ± 11.86	55.12 ± 11.44	48.57 ± 11.52	0.001
BMI (kg/m^2^)	24.17 ± 3.91	24.63 ± 4.28	23.94 ± 3.73	0.314
Smoking (yes)	28/152 (18.66%)	12/49 (21.79%)	16/103 (14.86%)	0.301
eGFR (mL/min/1.73 m^2^)	45.41 ± 25.25	32.92 ± 17.54	51.35 ± 6.24	<0.001
hs-CRP (mg/L)	11.44 ± 19.12	14.65 ± 20.12	9.89 ± 18.53	0.153
Hb (mg/dL)	12.12 ± 2.21	11.39 ± 2.32	12.46 ± 2.08	0.005
Mg (mg/dL)	1.71 ± 0.25	1.68 ± 0.28	1.72 ± 0.22	0.39
Albumin (g/dL)	39.38 ± 5.11	37.23 ± 5.34	40.40 ± 4.69	<0.001
Proteinuria (mg/24 h)	902.66 ± 1280.35	1286.65 ± 1041.66	719.98 ± 1345.98	0.01
Glycemia (mg/dL)	97.70 ± 26.25	107.79 ± 35.65	92.89 ± 18.69	0.008
Tacrolimus	104/152 (68.42%)	31/49 (58.97%)	73/103 (78.38%)	0.45
CsA	39/152 (25.66%)	13/49 (29.49%)	26/103 (21.62%)	0.84
RAP	8/152 (5.26%)	4/49 (10.26%)	4/103 (0%)	0.26
Neo	26/152 (17.11%)	8/49 (21.79%)	18/103 (12.16%)	0.52
Hypertension	148/152 (97.375)	49/49 (100%)	99/103 (94.59%)	0.30
Betablockers	132/152 (86.84%)	48/49 (93.59%)	84/103 (79.73%)	0.004
Statins	91/152 (59.87%)	38/49 (61.54%)	53/103 (55.13%)	0.003
NTproBNP (pg/mL)	5326.32 ± 8926.94	10,904.27 ± 12,197.32	1864.134 ± 2859.32	<0.001
HDL cholesterol (mg/dL)	45.91 ± 12.07	39.27 ± 10.80	49.07 ± 11.38	<0.001
TG (mg/dL)	161.39 ± 89.12	183.12 ± 70.13	151.06 ± 95.44	0.038
TG/HDL cholesterol	3.96 ± 2.98	5.10 ± 2.80	3.42 ± 2.92	0.008
LDL/HDL cholesterol	2.87 ± 1.42	3.68 ± 1.72	2.48 ± 1.06	<0.001
TyG index	8.84 ± 0.55	9.08 ± 0.567	8.73 ± 0.51	<0.001
AIP	0.16 ± 0.25	0.29 ± 0.22	0.09 ± 0.24	<0.001
PLR	174.45 ± 0.101	190.68 ± 0.10	167.38 ± 0.099	0.25
NLR/albumin	0.09 ± 0.06	0.109 ± 0.07	0.085 ± 0.045	0.047
CRP/albumin	0.31 ± 0.52	0.41 ± 0.58	0.26 ± 0.49	0.087
NLR	3.55 ± 2.11	3.99 ± 2.84	3.34 ± 1.62	0.076
MetS	62/152 (40.79%)	33/49 (52.56%)	29/103 (28.38%)	<0.001
EDC	78/152 (51.32%)	31/49 (56.41%)	47/103 (45.95%)	0.05

Legend: BMI—body mass index, eGFR—glomerular filtration rate, Hb—hemoglobin, Mg—magnesium, CsA—ciclosporin A, RAP—rapamycin, Neo—neoplastic disorders, NTproBNP—N terminal pro-B-type natriuretic peptide, HDL cholesterol—high density lipoprotein cholesterol, TG—triglycerides, LDL cholesterol—low density lipoprotein cholesterol, TyG—triglyceride glucose index, AIP—atherogenic index of plasma, PLR—platelet to lymphocyte ratio, NLR—neutrophils to lymphocyte ratio, hs-CRP—C reactive protein, MetS—metabolic syndrome, EDC—echocardiographic damage composite.

**Table 5:** Cox regression analysis for MACO predictors.

Parameters	HR	95% CI	*p*
TyG_01	1.039	0.993–1.088	0.09
eGFR	0.979	0.966–0.992	0.002
Proteinuria	1.000	1.000–1.000	0.16
Age	1.014	0.989–1.039	0.27
Sex	0.967	0.599–1.559	0.89
hs-CRP	1.010	1.000–1.020	0.05

Legend: TyG—triglyceride glucose index, eGFR—glomerular filtration rate, hs-CRP—high sensitive C reactive protein.

**Table 6:** Cox regression analysis for MACCE predictors.

Parameters	HR	95% CI	*p*
TyG_01	1.102	1.043–1.164	0.001
eGFR	0.975	0.957–0.993	0.007
Proteinuria	1.000	1.000–1.000	0.938
Age	1.029	0.999–1.060	0.060
Sex	1.091	0.609–1.954	0.770
hs-CRP	1.000	0.985–1.014	0.969

Legend: TyG—triglyceride glucose index, eGFR—estimated glomerular filtration rate, hs-CRP—high specific C reactive protein.

## Data Availability

The data presented in this study are available on request from the corresponding author. The data are not publicly available due to ethical reasons.
